# Left Ventricular Global Longitudinal Strain Is Associated With Cardiovascular Outcomes in Patients Who Underwent Permanent Pacemaker Implantation

**DOI:** 10.3389/fcvm.2021.705778

**Published:** 2021-07-30

**Authors:** Dae-Young Kim, Purevjargal Lkhagvasuren, Jiwon Seo, Iksung Cho, Geu-Ru Hong, Jong-Won Ha, Chi Young Shim

**Affiliations:** Division of Cardiology, Severance Cardiovascular Hospital, Yonsei University College of Medicine, Seoul, South Korea

**Keywords:** pacemaker, left ventricular global longitudinal strain, left ventricular ejection fraction (LVEF), cardiomyopahty, outcome

## Abstract

**Background:** Patients who underwent permanent pacemaker (PM) implantation have a potential risk of left ventricular (LV) systolic dysfunction. However, assessment of LV ejection fraction (LVEF) shows a limited role in identifying subclinical LV systolic dysfunction and predicting cardiovascular (CV) outcomes.

**Methods:** We reviewed 1,103 patients who underwent permanent PM implantation between January 2007 and December 2017. After excluding patients who did not undergo echocardiograms before or after PM implantation and those with LV ejection fraction (LVEF) <50%, significant valve dysfunction, and history of cardiac surgery before PM implantation, 300 (67 ± 13 years, 119 men) were finally analyzed. LV mechanical function was assessed with LV global longitudinal strain (LV-GLS) using 2-dimensional speckle-tracking echocardiography. CV outcomes were defined as a composite of CV death and hospitalization for heart failure.

**Results:** At 44 ± 28 months after post-PM echocardiogram, 23 patients (7.7%) had experienced CV outcomes. Patients with CV outcomes were older and had more comorbidities and a lower baseline |LV-GLS| than those without CV outcomes. LV mechanical function worsened after PM implantation in patients with CV outcomes. The cut-off value of 11.2% in |LV-GLS| on post-PM echocardiogram had a better predictive value for CV outcomes (AUC; 0.784 vs. 0.647, *p* = 0.012). CV outcome in patients with |LV-GLS| <11.2% was worse than that in those with |LV-GLS| ≥ 11.2% (log-rank *p* < 0.001). Multivariate Cox model revealed that reduced |LV-GLS| was independently associated with CV outcomes.

**Conclusions:** Pacing deteriorates LV mechanical function. Impaired LV-GLS is associated with poor CV outcomes in patients who underwent PM implantation.

## Introduction

Patients who have undergone permanent pacemaker (PM) implantation have a potential risk of left ventricular (LV) systolic dysfunction (1–3). PM-induced cardiomyopathy (PMIC) is generally defined as a decrease in LV systolic function after right ventricular (RV) pacing with no other independent triggering factors (4) and is associated with worse cardiovascular (CV) outcomes (5). Theoretically, increasing RV pacing causes LV mechanical dyssynchrony and consequently leads to a decrease in LV ejection fraction (LVEF) and CV events related to worsening heart failure (6–8). Although LVEF is the most widely used echocardiographic parameter representing LV systolic function, its measurement can be less reliable in patients with PM because of LV dyssynchronous contraction (9). Moreover, assessment of LVEF shows a limited role in identifying subclinical LV systolic dysfunction, both in patients at risk of PMIC and in the early stages of PMIC.

Assessment of LV mechanical function using LV-global longitudinal strain (LV-GLS) by 2-dimensional speckle-tracking echocardiography can detect subclinical LV dysfunction in the early stages of cardiomyopathy to provide prognostic information (10–12). A previous study demonstrated that LV-GLS could provide better risk stratification than could LVEF among patients with LV dyssynchrony caused by left bundle branch block (13). Therefore, we hypothesized that there would be a significant relationship between LV-GLS measured by 2D speckle-tracking echocardiography and the occurrence of CV outcomes in patients with permanent PM.

## Materials and Methods

### Study Population

A total of 1,103 patients who underwent permanent PM implantation at a single tertiary hospital between January 2007 and December 2017 was identified retrospectively. Among them, we selected patients who underwent both baseline transthoracic echocardiography within 1 year before PM implantation and follow-up echocardiography between 6 months and 5 years after PM implantation. We excluded patients who had overt LV systolic dysfunction (LVEF <50%) before PM implantation, had at least a moderate degree of any valve dysfunction on baseline echocardiography, had a history of cardiac surgery, acute myocardial infarction within 3 months before PM implantation, and single-lead right atrial PM. All patients who had cardiovascular events in time between baseline and post-implantation echocardiogram were also excluded from this study cohort. Finally, 300 patients were included in the analysis. Patients' clinical data, medications, PM characteristics, echocardiographic characteristics, and clinical outcomes were reviewed retrospectively. The study was approved by the Institutional Review Board of Yonsei University Health System (approval number: 4-2020-0032) and conducted according to the Declaration of Helsinki.

### Follow-Up and Outcomes

Patients were scheduled to visit the PM clinic every 6 months after PM implantation. Follow-up data, including pacing percentage and clinical events, were obtained by reviewing medical records. Data of pacing percentage was gathered at the time of the first interrogation after 2 months of PM implantation. Based on the echocardiographic data between 6 months and 5 years after PM implantation, PMIC was defined as a ≥10% decrease in LVEF compared with baseline echocardiography with resultant LVEF <50% (14). We reviewed all medical records after PM implantation through total follow-up periods, and patients who had an event of myocardial infarction, acute coronary syndrome, and who had severe valvular stenosis/regurgitation on post-PM echocardiogram were excluded from later analysis. CV outcomes were defined as a composite of admission for heart failure and CV death and the event was included only after the time of post-PM echocardiography. Admission of heart failure was defined when following conditions were met: the patient's symptom of dyspnea at least 3 of New York Heart Association (NYHA) class, required medication such as diuretics or vasodilators, elevated N-terminal pro-brain natriuretic peptide (NT-proBNP), and pulmonary edema or pleural effusion in chest X-ray. CV death was defined as the cause of the death was acute myocardial infarction, heaft failure, sudden cardiac death, lethal ventricular arrhythmia, or stroke, and we determined the CV death by review the patient's medical record and the death certificate. If a patient had at least two clinical outcomes, the first event was included for outcome analyses.

### Echocardiography

Standard 2D and Doppler measurements were performed using a standard ultrasound machine (Vivid E9; GE Medical Systems, Chicago, IL; Philips iE33; Philips Healthcare, Netherlands) with a 2.5–3.5 MHz probe. Standard echocardiographic measurements were performed according to recommendations from the European association of cardiovascular imaging (15). LVEF was measured using the biplane Simpson's method in apical four- and two-chamber views. Left atrial volume index was measured by the biplane method in both the apical four- and two-chamber views and indexed on the body surface area. The severity of tricuspid regurgitation was graded with multi-parametric methods. (16).

### Speckle-Tracking Echocardiography

**Figure 1:**
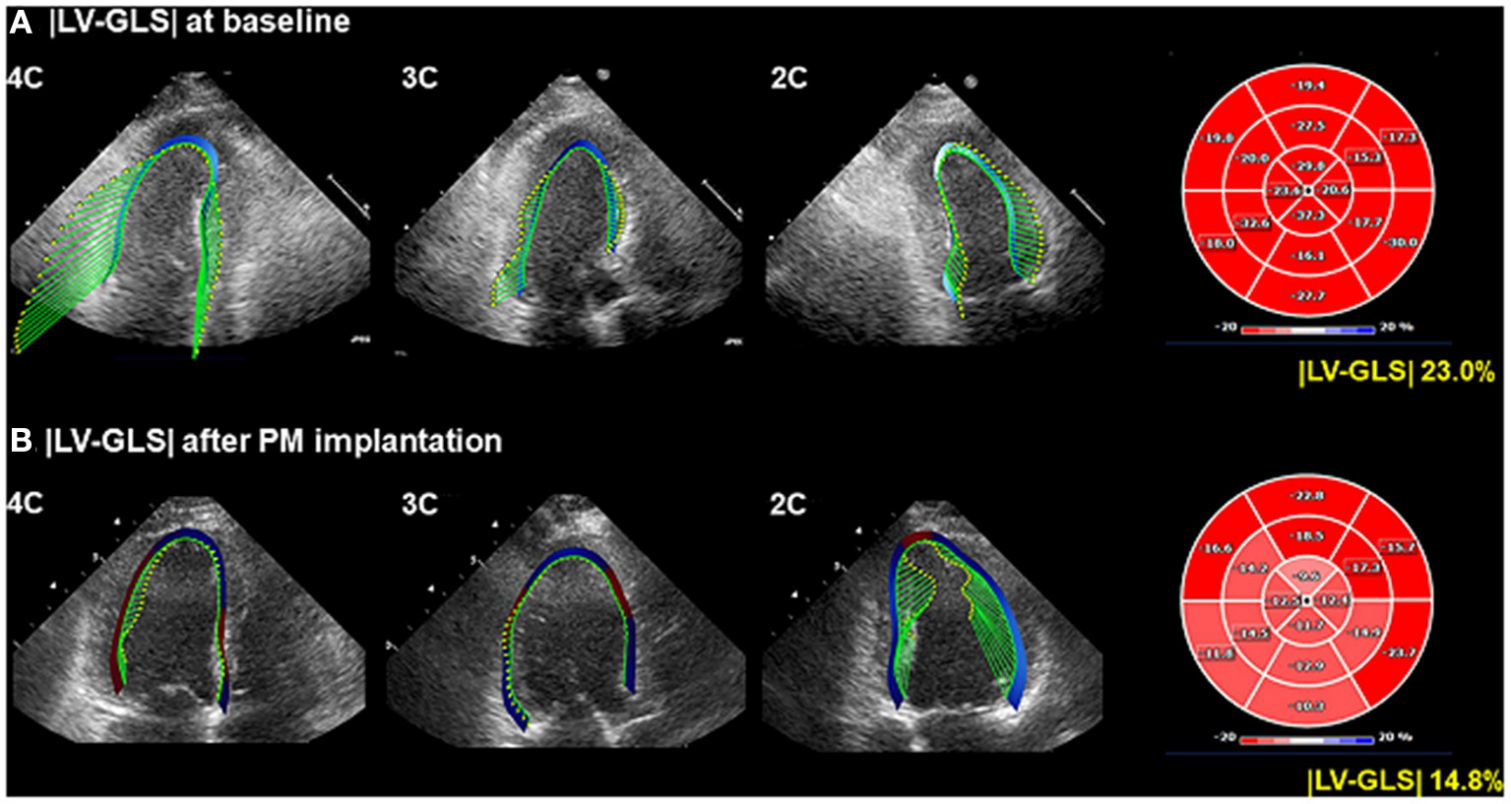
Representative LV-GLS measurements. **(A)** LV-GLS was calculated from three standard apical views at baseline echocardiogram (left). The absolute value of LV-GLS was 23.0% (right). **(B)** LV-GLS was assessed after PM implantation. |LV-GLS| was 14.8% (right). LV-GLS, left ventricular global longitudinal strain; PM, pacemaker.

The two-dimensional images from both baseline echocardiogram and echocardiogram after PM implantation were used for analyses of LV mechanical function. Three apical four-, three-, and two-chamber images for each echocardiographic study were stored and exported to the off-line data storage device, and speckle-tracking echocardiography was performed using a vendor-independent software package (TomTec software; Image Arena 4.6, Munich, Germany) as described previously (17). After that, LV-GLS and segmental longitudinal strain values were measured in a blinded method from the clinical data by experienced cardiologists, according to the practical guidance in assessing strain (18, 19). LV endocardial borders were traced at the end-diastolic and end-systolic frames in each apical view. TomTec software tracked the speckle on three endocardial borders during the whole cardiac cycle. For analysis of segmental strain, we used the 16-segment model that divided the base and mid area into six segments (antero-septum, anterior, antero-lateral, infero-lateral, inferior, and infero-septal) and the apex into four segments (septal, inferior, lateral, and anterior), and LV-GLS was calculated by averaging the values at each segmental level as mentioned above. |LV-GLS| was defined as the absolute value of LV-GLS (removing the conventional negative value of LV-GLS data) (Figure 1). The LV-global circumferential strain (GCS) was calculated by averaging the values of segmental circumferential strains from the parasternal short-axis view in the basal-, mid-, and apical-LV levels. |LV-GLS| was defined as the absolute value of LV-GCS. We randomly selected 20 patients from the study population and analyzed the intra- and inter-observer reproducibility of LV-GLS measurement by Bland–Altman analysis. The intra-class correlation coefficients for |LV-GLS| were 0.987 and 0.963 for intra- and inter-observer variation, respectively. The Bland–Altman analysis showed the limits of agreement (LOA) across a broad range of LV-GLS values; the bias for intra- and inter-observer measurements of LV-GLS was 0.47% (range: −0.91 to −0.04%, 95% LOA) and 0.37% (range: −0.71 to 0.78%), respectively.

### Statistical Analysis

Continuous variables are presented as mean ± standard deviation (SD), and categorical variables are presented as frequency and percentage. Comparisons of baseline clinical and echocardiographic parameters between the two groups were analyzed using Student's *t*-test for continuous data and chi-square (χ2) and Fisher's exact test for categorical data. Correlations between pacing percentage and echocardiographic variables including the strain of each segment were obtained using simple linear regression analysis and multiple linear regression analysis for adjusting other confounding factors. Predictive values of LV-GLS for CV outcomes were calculated using receiver operating characteristic (ROC) analysis. Clinical outcomes were constructed using Kaplan–Meier methods, and comparisons among groups were performed using a log-rank test. The predictors of CV outcomes were evaluated using multivariate nested Cox proportional hazard regression models. The independence of |LV-GLS| was examined using three models. Initially, three subgroups divided by |LV-GLS| tertile were included in the Cox model as a covariate, with adjustment for age and sex. Then, chronic kidney disease and coronary artery disease were included in the second model. Finally, LVEF and left atrial volume index were included in the last model. Significant differences were considered at *P* <0.05. All statistical analyses were performed using SPSS 25.0 software (IBM Corp., Armonk, NY), and Medcalc statistical package (Medcalc software, Mariakerke, Belgium) was used for comparison of ROC curves.

## Results

### Baseline Characteristics

During a mean 44 ± 28 months of follow-up after post-PM echocardiography, 23 of 300 patients (7.7%) experienced CV events. Clinical characteristics, medications, and data related to PM in patients with or without CV outcomes are presented in Table 1. Patients with CV outcomes were older and had a higher prevalence of chronic kidney disease, coronary artery disease than did those without CV outcomes. More heart failure medications including renin-angiotensin-aldosterone system blockade and diuretics were used by patients with CV outcomes compared to those without CV outcomes. The ventricular lead position and pacing percentage were not different between the groups.

**Table 1:** Baseline characteristics.

	**Total (*n* = 300)**	**Without CV outcomes (*n* =277)**	**With CV outcomes (*n* = 23)**	***P*-value**
Age, years	67.1 ± 13.2	66.2 ± 13.8	74.5 ± 9.9	0.005
Male sex, *n* (%)	119 (39.7)	107 (38.6)	12 (52.2)	0.202
BMI, kg/m^2^	24.3 ± 3.6	24.3 ± 3.6	25.0 ± 3.6	0.357
Hypertension, *n* (%)	171 (57.0)	155 (56.0)	16 (69.6)	0.205
Diabetes mellitus, *n* (%)	61 (20.3)	54 (19.5)	7 (30.4)	0.210
CKD, *n* (%)	22 (7.3)	16 (5.8)	6 (26.1)	<0.001
AF, *n* (%)	54 (18.0)	48 (17.3)	6 (26.1)	0.293
CAD, *n* (%)	39 (13.0)	30 (10.8)	9 (39.1)	<0.001
HFpEF, *n* (%)	5 (1.7)	4 (1.4)	1 (4.3)	0.296
Medications, *n* (%)				
RAAS blockers	141 (47.0)	122 (44.0)	19 (82.6)	<0.001
Beta blockers	39 (13.0)	36 (13.0)	3 (13.0)	0.995
CCB	69 (23.0)	64 (23.1)	5(21.7)	0.881
Diuretics	78 (26.0)	68 (24.5)	10 (43.5)	0.047
Statin	89 (29.7)	81 (29.2)	8 (34.8)	0.576
V lead position, *n* (%)				
Apex	273 (91.0)	250 (90.3)	23 (100.0)	0.117
Septum	27 (9.0)	27 (9.7)	0 (0.0)	
Pacing percentage, %	60.4 ± 42.4	61.3 ± 42.3	49.7 ± 42.6	0.211

*CV, cardiovascular; BMI, body mass index; CKD, chronic kidney disease; AF, atrial fibrillation; CAD, coronary artery disease; HFpEF, heart failure with preserved ejection fraction; RAAS, renin-angiotensin-aldosterone system; CCB, calcium channel blockers; V, ventricular*.

Baseline and post-PM echocardiographic variables are presented in Table 2. On baseline echocardiogram, patients with CV outcomes showed lower e' and S' velocities, higher E/e', and lower |LV-GLS| than did those without CV outcomes. On post-PM echocardiogram, more dilated chambers, relatively low LVEF (even though mean LVEF was within the normal range), higher LV mass index, lower S' velocity, higher E/e', and lower |LV-GLS| and |LV-GCS| were shown in patients with CV outcomes compared to patients without CV outcomes. Timing of post-PM echocardiogram after PM implantation was not different between the groups.

**Table 2:** Echocardiographic characteristics.

	**Total (*n* = 300)**	**Without CV outcomes (*n* = 277)**	**With CV outcomes (*n* = 23)**	***P*-value**
**Baseline echocardiogram**				
LVEDD, mm	50.3, 4.7	50.2, 4.6	51.4, 5.5	0.250
LVESD, mm	32.3, 4.3	32.2, 4.2	33.7, 5.4	0.120
LVEF. %	67.8, 6.8	67.9, 6.7	66.0, 7.5	0.191
LV mass index, g/m^2^	104.4, 22.5	103.8, 22.3	112.1, 25.0	0.130
LA volume index, ml/m^2^	35.8, 12.6	35.4, 12.5	40.4, 14.0	0.068
e' velocity, cm/s	6.1, 2.4	6.2, 2.5	4.7, 1.5	0.012
S' velocity, cm/s	6.7, 1.6	6.8, 1.6	5.8, 1.4	0.007
E/e'	13.7, 6.3	13.3, 5.9	19.4, 8.7	0.007
|LV-GLS|, %	21.0, 5.3	21.2, 5.3	18.5, 4.7	0.016
|LV-GCS|, %	29.1, 7.0	29.2, 7.0	27.6, 6.8	0.292
**Post-PM echocardiogram**				
Time after PM implantation, years	2.2, 1.2	2.2, 1.2	2.4, 1.4	0.730
LVEDD, mm	49.6, 5.1	49.4, 5.0	51.7, 6.2	0.035
LVESD, mm	33.4, 5.7	33.1, 5.5	36.4, 7.5	0.047
LVEF. %	62.2, 10.3	62.7, 9.7	55.1, 14.1	0.018
PMIC, n (%)	32 (10.7)	22 (7.9)	10 (43.5)	<0.001
LV mass index, g/m^2^	101.4, 25.3	100.0, 24.6	120.0, 27.7	<0.001
LA volume index, ml/m^2^	34.8, 13.4	34.0, 12.9	44.8, 15.3	<0.001
e' velocity, cm/s	5.3, 1.9	5.3, 1.9	5.0, 1.3	0.476
S' velocity, cm/s	5.9, 1.4	5.9, 1.4	4.9, 0.9	<0.001
E/e'	13.4, 5.9	13.0, 5.5	19.1, 8.4	0.007
Significant TR (≥grade 2)	18 (6.0)	17 (6.1)	1 (4.3)	0.728
|LV-GLS|, %	16.6, 5.3	17.0, 5.0	11.2, 5.5	<0.001
|LV-GCS|, %	24.5, 7.1	24.9, 6.9	19.1, 7.7	<0.001

*CV, cardiovascular; LVEDD, left ventricular end-diastolic dimension; LVESD, left ventricular end-systolic dimension; LVEF, left ventricular ejection fraction; LV, left ventricle; LA, left atrium; e', early diastolic mitral annular tissue velocity; S', systolic mitral annular tissue velocity; E/e', ratio of early diastolic mitral inflow velocity to early diastolic mitral annular tissue velocity; |LV-GLS|, absolute value of left ventricular global longitudinal strain; |LV-GCS|, absolute value of left ventricular global circumferential strain; PM, pacemaker; PMIC, pacemaker-induced cardiomyopathy; TR, tricuspid regurgitation*.

### Relationship Between RV Pacing and LV Mechanical Dysfunction

The correlations between RV pacing percentage on the first PM interrogation results and the parameters on post-PM echocardiogram are presented in Table 3. As RV pacing percentage increased, functional and structural deterioration was observed. The correlation between RV pacing percentage and |LV-GLS| was significant (*r* = 0.257, *p* <0.001) compared to that of other global functional parameters and this phenomenon was strengthened especially with RV apical pacing, compared with RV septal pacing (*p* < 0.001 vs. *p* = 0.248). In terms of LV segmental strain, RV pacing percentage was significantly associated with impaired strain at all segments in the LV apex and at inferior and septal segments in the mid-LV. Multiple linear regression analysis revealed that RV pacing percentage was independently associated with impaired |LV-GLS| and |Apical septal strain| after adjusting for age, sex, HTN, and DM in all the study subjects (both *p* < 0.001) (Supplementary Table 1). When compared the subgroup with RV pacing percentage divided by 50%, the subgroup with the RV pacing under 50% had better event-free survival than those who were not (log rank p = 0.047) (Supplementary Figure 1).

**Table 3:** Simple correlations between pacing percentage and echocardiographic variables after PM implantation.

		**Correlation coefficient**	***P*-value**
LVEDD, mm		0.162	0.005
LVESD, mm		0.189	0.001
LVEF. %		−0.210	<0.001
LV mass index, g/m^2^		0.116	0.048
LA volume index, ml/m^2^		0.100	0.083
e' velocity, cm/s		−0.138	0.021
S' velocity, cm/s		−0.202	0.001
E/e'		0.013	0.833
|LV-GLS|, %		−0.257	<0.001
|LV-GCS|, %		−0.198	0.001
LV segmental longitudinal strain			
Base	Antero-septum	0.093	0.109
	Anterior	0.050	0.385
	Antero-lateral	0.073	0.207
	Infero-lateral	0.076	0.191
	Inferior	0.126	0.029
	Infero-septal	0.127	0.028
Mid LV	Antero-septum	0.183	0.001
	Anterior	0.081	0.164
	Antero-lateral	0.133	0.021
	Infero-lateral	0.058	0.316
	Inferior	0.270	<0.001
	Infero-septal	0.182	0.002
Apex	Septal	0.333	<0.001
	Anterior	0.236	<0.001
	Lateral	0.316	<0.001
	Inferior	0.271	<0.001

*LVEDD, left ventricular end-diastolic dimension; LVESD, left ventricular end-systolic dimension; LVEF, left ventricular ejection fraction; LV, left ventricle; LA, left atrium; e', early diastolic mitral annular tissue velocity; S', systolic mitral annular tissue velocity; E/e', ratio of early diastolic mitral inflow velocity to early diastolic mitral annular tissue velocity; |LV-GLS|, absolute value of left ventricular global longitudinal strain; |LV-GCS|, absolute value of left ventricular global circumferential strain*.

### Occurrence of PMIC and CV Outcomes Based on LV-GLS Levels

**Figure 2:**
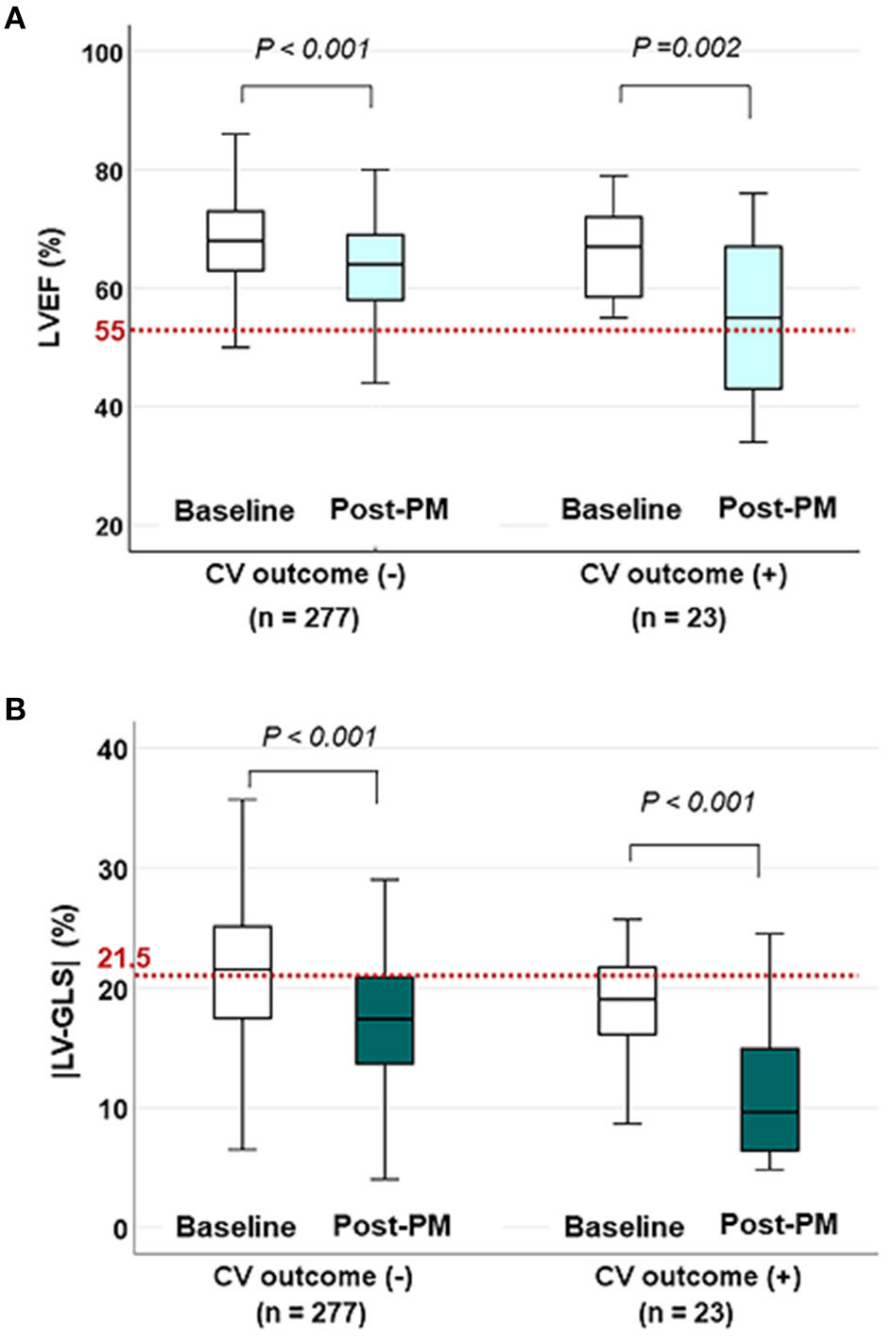
The changes in LVEF and |LV-GLS| according to CV outcomes on baseline and post-PM echocardiogram. **(A)** LVEF decreased after PM implantation in both groups, but the mean values were within the normal range (red dotted line) even in patients with CV outcomes. **(B)** |LV-GLS| in patients with CV outcomes decreased more noticeably than in patients without CV outcomes under normal values (red dotted line). LVEF, left ventricular ejection fraction; CV, cardiovascular; |LV-GLS|, absolute value of left ventricular global longitudinal strain; PM, pacemaker.

The changes in LVEF and |LV-GLS| according to the CV outcomes at the baseline echocardiogram and post-PM follow-up study are shown in Figure 2. LVEF and |LV-GLS| were decreased after PM implantation in the group without CV outcomes and the group with CV outcomes. However, LVEF after PM implantation maintained normal values in both groups, whereas |LV-GLS| decreased more noticeably in patients with CV outcomes. When compared the subgroup which was divided by the degree of |LV-GLS| change, the subgroup with the |LV-GLS| reduction under 10% had better event-free survival than those who were not (log rank *p* = 0.035) (Supplementary Figure 2).

**Figure 3:**
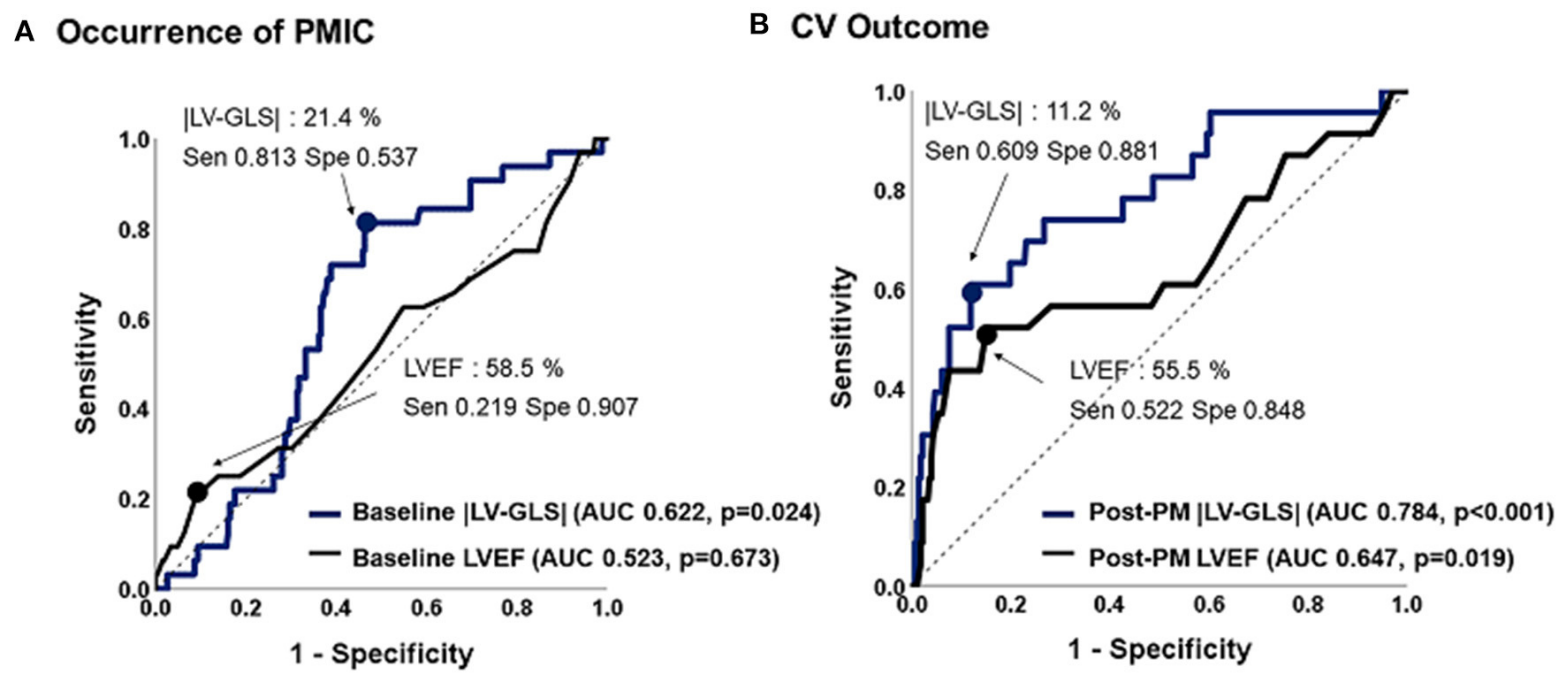
Predictive values of |LV-GLS| and LVEF for occurrence of PMIC and CV outcome. **(A)** The |LV-GLS| on baseline echocardiogram revealed significant predictive value for occurrence of PMIC. **(B)** The |LV-GLS| on post-PM echocardiogram showed a better predictive value for CV outcomes than did LVEF. |LV-GLS|, absolute value of left ventricular global longitudinal strain; LVEF, left ventricular ejection fraction; PMIC, pacemaker-induced cardiomyopathy; PM, pacemaker; CV, cardiovascular.

ROC analysis of predictive values of LVEF and |LV-GLS| for PMIC and CV outcomes are shown in Figure 3. The |LV-GLS| on baseline echocardiogram revealed a significant predictive value for PMIC (area under the curve, AUC: 0.622, *p* = 0.024, cut-off 21.4%, sensitivity 81.3%, specificity 53.7%) after PM implantation. In terms of CV outcomes, the cut-off value of 11.2% in |LV-GLS| on post-PM echocardiogram showed a better predictive value than did LVEF (AUC; 0.784 vs. 0.647, *p* = 0.012), with acceptable sensitivity (60.9 %) and specificity (88.1%).

**Figure 4:**
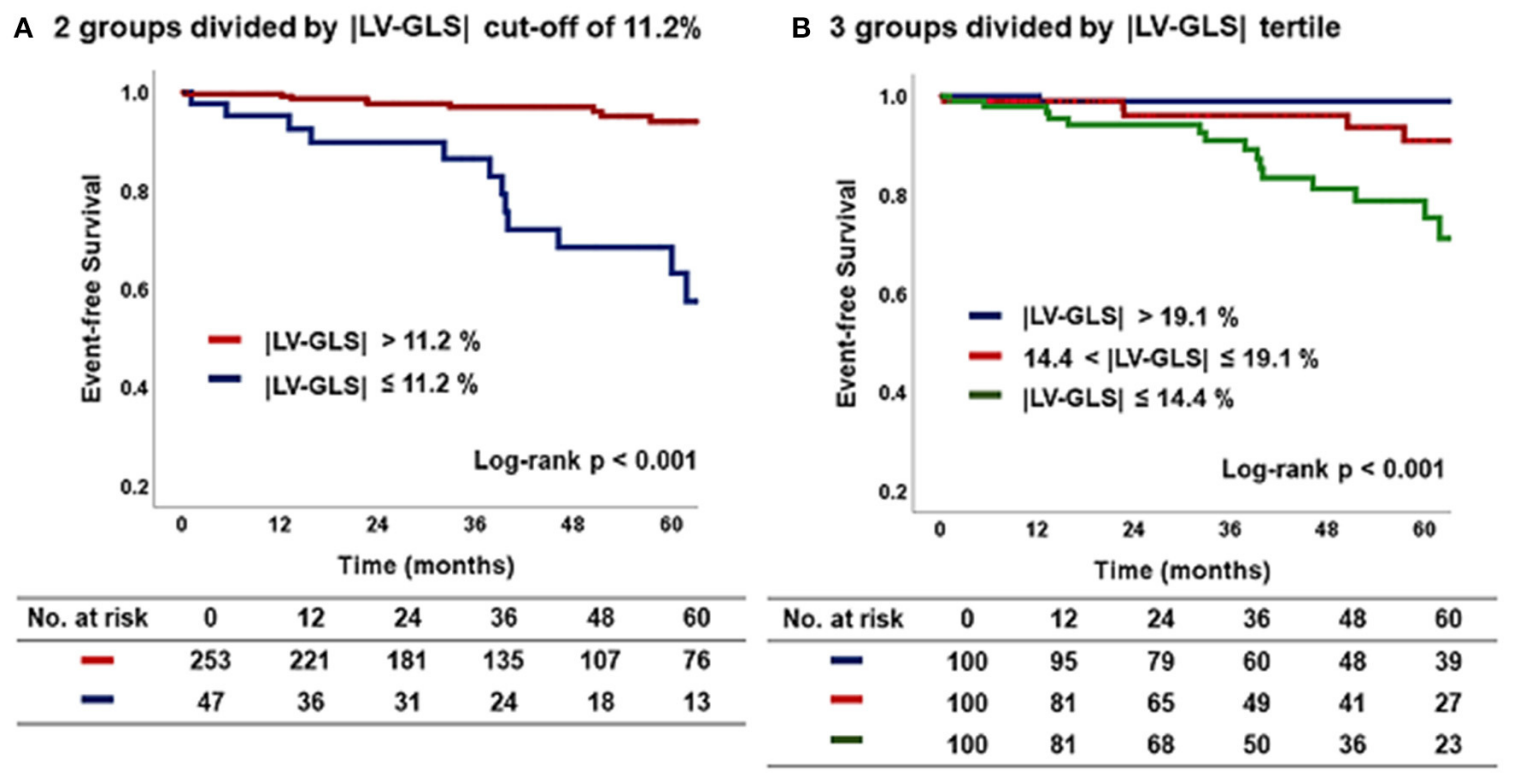
Kaplan–Meier curve according to |LV-GLS| on post-PM echocardiogram. **(A)** The group of |LV-GLS| ≤ 11.2% revealed a significantly worse CV outcome than the other group (log-rank *p* < 0.001). **(B)** The lowest |LV-GLS| group revealed a significantly worse CV outcome than the others (log-rank *p* < 0.001). |LV-GLS|, absolute value of left ventricular global longitudinal strain; PM, pacemaker; CV, cardiovascular.

Figure 4 shows the Kaplan–Meier survival curves for the two groups divided by an |LV-GLS| of 11.2 % and for three subgroups divided by |LV-GLS| tertile. At a mean 44 ± 28 months of follow-up after post-PM echocardiogram, the lowest |LV-GLS| group revealed a significantly worse CV outcome than did the others (log-rank *p* < 0.001). In multivariate nested Cox proportional hazard models, the lower |LV-GLS| showed an independent association with poor CV outcomes in age and sex adjusted analysis (hazard ratio, HR: 14.8; 95% confidence interval, CI: 1.93–112.70, *p* = 0.009). Multivariate models including comorbidities and conventional echocardiographic variables also revealed that the lower |LV-GLS| had a statistically significant association with poor CV outcomes (Model 2; HR: 15.18; CI: 1.96–117.61; *p* = 0.009) (Model 3; HR: 13.97; CI: 1.72–113.39; *p* = 0.014) (Table 4).

**Table 4:** Cox regression analysis for CV outcomes.

	**Model 1**		**Model 2**		**Model 3**	
	**Hazard ratio [95% CI]**	***P*-value**	**Hazard ratio [95% CI]**	***P*-value**	**Hazard ratio [95% CI]**	***P*-value**
|LV-GLS| > 19.1%	Reference		Reference		Reference	
14.4 < |LV-GLS| ≤ 19.1%	5.80 [0.68~49.84]	0.109	4.06 [0.46~35.66]	0.207	4.59 [0.52~40.13]	0.169
|LV-GLS| ≤ 14.4%	14.8 [1.93~112.70]	0.009	15.18 [1.96~117.61]	0.009	13.97 [1.72~113.39]	0.014

*Model 1: Adjusted by age and sex.*

*Model 2: Adjusted by age, sex, CKD, and CAD.*

*Model 3: Adjusted by age, sex, CKD, CAD, LVEF, and LA volume index.*

*CI, confidence interval; CKD, chronic kidney disease; CAD, coronary artery disease; LVEF, left ventricular ejection fraction; LA, left atrium; |LV-GLS|, absolute value of left ventricular global circumferential strain*.

## Discussion

The principal findings of the present study were as follows: (1) RV pacing deteriorated global and segmental LV mechanical function, (2) |LV-GLS| at baseline echocardiogram showed a good predictive value for PMIC after PM implantation, (3) |LV-GLS| at post-PM echocardiogram revealed a better predictive value of CV outcomes than did LVEF, and (4) reduced |LV-GLS| was independently associated with poor CV outcomes in patients with permanent PM. These results imply that, for patients who undergo permanent PM implantation, serial assessments of LV-GLS by speckle-tracking echocardiography before and after PM implantation are beneficial for early detection of PMIC and prediction of clinical outcomes.

### Usefulness of LV-GLS in Patients Who Underwent PM Implantation

As the population ages, not only is the number of patients receiving permanent PM implantation increasing, but so is the occurrence of heart failure after PM implantation. (20, 21). PMIC has emerged as one of the most important complications of PM implantation, and its incidence is increasing, increasing the need to upgrade to cardiac resynchronization therapy (4). PMIC is usually defined as a decrease of LVEF, but there are many limitations in predicting PMIC before LV systolic dysfunction. The present study demonstrated that the cut-off value of 21.4 % in |LV-GLS| on baseline echocardiogram before PM implantation revealed a significant predictive value for occurrence of PMIC after PM implantation, but LVEF did not. In a previous study, Ahmed et al. showed changes in |LV-GLS| and LVEF after PM implantation in 55 patients. (22) 1 month after PM implantation, |LV-GLS| was significantly reduced from 16.3 ± 0.5 to 12.6 ± 0.9% in patients with PMIC compared to baseline. At 12 months of follow-up, |LV-GLS| impairment was reported as 11.9 ± 2.5%. This showed the sensitivity of LV-GLS in predicting the development of PMIC. However, the study included a small cohort of 55 patients, and the follow-up periods were relatively short for estimating CV outcomes. Moreover, LV-GLS was evaluated 1 month after PM implantation, and additional echocardiographic studies are required. On the other hand, the present study showed the predictive value of LV-GLS in the occurrence of PMIC, and this was assessed before PM implantation in a relatively large number of patients. In another study, Babu et al. demonstrated that 3D echocardiography with |LV-GLS| analysis played a role in predicting PMIC in a total of 36 patients (23). A decline in |LV-GLS| of 18.0% to 13.9% was noted at 6-months after PM implantation, whereas a decline in LVEF of 57.8% to 54.5% was noted after PM implantation. This study was conducted in a small population of 36 patients with short follow-up periods. Accordingly, the present study demonstrates the usefulness of LV-GLS for predicting PMIC occurrence and clinical outcome in patients with both baseline and post-PM echocardiography and sufficient regular clinical follow-up for an average of 44 months after post-PM echocardiography.

### Pacing Percentage and LV Mechanical Dysfunction

It is well known that the incidence of heart failure in patients who underwent PM implantation is associated with RV pacing, and that a higher RV pacing percentage results in a higher incidence of CV events (24). In this study, it was confirmed that, as RV pacing percentage increased, the global LV mechanical function evaluated by |LV-GLS| decreased, and it was more obvious when the analysis was performed divided by RV pacing site. Previous studies also tried to show the relationship between pacing percentage and LV-GLS; however, mainly due to the small number of study subjects, they failed to show a linear correlation (22, 25). Another strength of the present study is that it comprehensively shows LV regional mechanical dysfunction by pacing through segmental strain analysis. As pacing percentage increased, the absolute value of longitudinal strain in some specific segments decreased. The specific segments that were highly affected by pacing were those in the entire LV apex. The correlation weakened toward the mid-LV and base, but the absolute value of segmental strain in the inferior segment and infero-septal segment significantly decreased in proportion to pacing percentage. These results can be interpreted based on the pathophysiology of PMIC. During RV pacing, conduction of an electrical wave passes through the myocardium, which is adjacent to the PM lead (6, 26). Therefore, there were significant decreases in strain in the regions close to the pacing area (27). This phenomenon results in dyssynchronous motion of LV. In patients with dyssynchronous motion due to higher RV pacing burden and subsequent lower LVEF, an upgrade from PM to cardiac resynchronization therapy might be considered to improve LV mechanical dysfunction and to treat heart failure (5). Considering the cut-off value of |LV-GLS| of post-PM implantation as 11.2%, patients with |LV-GLS| under 11.2% on post-PM implantation echocardiography are recommended to adjust the PM pacing parameters to reduce the pacing percentage or consider to converse to cardiac resynchronization therapy, with aggressive heart failure medication. In patients with |LV-GLS| in the gray zone between 11.2 to 20%, regular echocardiography follow-up to identify whether |LV-GLS| decreases under 11.2% and manage several risk factors regarding heart failure are required.

### Limitations

This study has several limitations. First, this study was retrospectively designed and comprised patients who were followed up by regular visits. The interval of follow-up echocardiogram after PM implantation was not concordant in all patients. To minimize this limitation, the timing of follow-up echocardiogram was limited to between 6 months and 5 years after PM implantation to evaluate the predictability of future CV events of LV-GLS. Nevertheless, the population might be biased, and it is possible that the occurrence of clinical events was underestimated. Second, echocardiographic examinations of patients were not performed using the same equipment, which could have produced inconsistency of echocardiographic parameters, especially LV-GLS. However, we used vendor-independent software and tried to minimize the error of measurement by expert operators. Third, the |LV-GLS| value for predicting PMIC on the baseline echocardiogram before PM implantation was 21.4%, which was within the normal range. According to previous results of a head-to-head comparison of LV-GLS among vendors reported by the European Association of Cardiovascular Imaging/American Society of Echocardiography, the average |LV-GLS| values measured by TomTec software was 21.5%, trending higher than other software measures. (28). Also, there is a possibility that |LV-GLS| was high because of a compensatory increase in stroke volume due to bradycardia in some patients. As a result, we suggest that, if |LV-GLS| is lower than the normal reference value before PM implantation, close clinical and echocardiographic follow-up should be performed after PM implantation considering the risk of PMIC.

## Conclusion

After PM implantation, there were significant regional and global changes in LV mechanical function. On post-PM echocardiogram, reduced |LV-GLS| rather than LVEF is associated with poor CV outcome. Assessments of LV-GLS by speckle-tracking echocardiography before and after PM implantation are beneficial for early detection of LV mechanical dysfunction and prediction of CV outcomes.

## Data Availability Statement

The original contributions presented in the study are included in the article/Supplementary Material, further inquiries can be directed to the corresponding author.

## Ethics Statement

The studies involving human participants were reviewed and approved by Yonsei University Health System. Written informed consent for participation was not required for this study in accordance with the national legislation and the institutional requirements. Written informed consent was not obtained from the individual(s) for the publication of any potentially identifiable images or data included in this article.

## Author Contributions

D-YK and CS contributed to the concept and design of this study. D-YK, PL, JS, IC, G-RH, J-WH, and CS contributed to acquisition, analysis, and interpretation of the data. D-YK and CS contributed to drafting of the manuscript and statistical analysis. G-RH, J-WH, and CS contributed to revision and finalize the manuscript. All authors contributed to the article and approved the submitted version.

## Conflict of Interest

The authors declare that the research was conducted in the absence of any commercial or financial relationships that could be construed as a potential conflict of interest.

## Publisher's Note

All claims expressed in this article are solely those of the authors and do not necessarily represent those of their affiliated organizations, or those of the publisher, the editors and the reviewers. Any product that may be evaluated in this article, or claim that may be made by its manufacturer, is not guaranteed or endorsed by the publisher.

## Supplementary Material

Supplementary Table 1 Multiple linear regression analysis for post-PM |LV-GLS| and |Apical septal strain|. |LV-GLS|, absolute value of left ventricular global longitudinal strain; |Apical septal strain|, absolute value of left ventricular apical septal strain; RV, right ventricle; HTN, hypertension; DM, diabetes mellitus.

Supplementary Figure 1 Kaplan-Meier curve on 2 subgroups according to the RV pacing percentage divided by 50%. The subgroup with RV pacing under 50% had better event-free survival than those who were not (log rank p = 0.047). RV; right ventricle.

Supplementary Figure 2 Kaplan-Meier curve on 2 subgroups according to the degree of |LV-GLS| change between baseline and post-PM echocardiogram. The subgroup with the |LV-GLS| reduction under 10% had better event-free survival than those who were not (log rank p = 0.035). |LV-GLS|; absolute value of left ventricular global longitudinal strain.
