# Supplementary material for: Left Ventricular Global Longitudinal Strain Is Associated With Cardiovascular Outcomes in Patients Who Underwent Permanent Pacemaker Implantation
Source: Front Cardiovasc Med. 2021 Jul 30;8:705778. doi: 10.3389/fcvm.2021.705778 (PMC8363313; doi:10.3389/fcvm.2021.705778)
Supplement: Supplementary Table 1 — Multiple linear regression analysis for post-PM |LV-GLS| and |Apical septal strain|. |LV-GLS|, absolute value of left ventricular global longitudinal strain; |Apical septal strain|, absolute value of left ventricular apical septal strain; RV, right ventricle; HTN, hypertension; DM, diabetes mellitus. [file Table_1.docx]

**Supplement table 1. Multiple linear regression analysis for post-PM |LV-GLS| and |Apical septal strain|**

|  | **\|LV-GLS\|** | | **\|Apical septal strain\|** | |
| --- | --- | --- | --- | --- |
|  | Coefficient | P-value | Coefficient | P-value |
| RV pacing percentage (%) | -0.242 | <0.001 | -0.327 | <0.001 |
| Age (years) | -0.135 | 0.026 | -0.114 | 0.058 |
| Male sex | -0.049 | 0.369 | -0.050 | 0.360 |
| HTN | -0.079 | 0.199 | -0.005 | 0.931 |
| DM | -0.124 | 0.029 | -0.068 | 0.230 |

|LV-GLS|, absolute value of left ventricular global longitudinal strain; |Apical septal strain|, absolute value of left ventricular apical septal strain; RV, right ventricle; HTN, hypertension; DM, diabetes mellitus
