# Supplementary figures and images for: Left Ventricular Global Longitudinal Strain Is Associated With Cardiovascular Outcomes in Patients Who Underwent Permanent Pacemaker Implantation
Source: Front Cardiovasc Med. 2021 Jul 30;8:705778. doi: 10.3389/fcvm.2021.705778 (PMC8363313; doi:10.3389/fcvm.2021.705778)

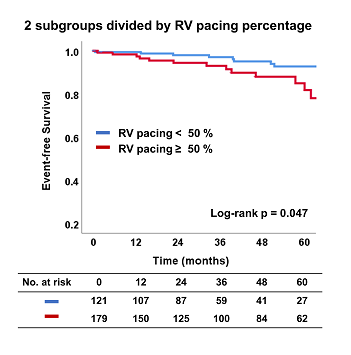

Supplement: Supplementary Figure 1 — Kaplan-Meier curve on 2 subgroups according to the RV pacing percentage divided by 50%. The subgroup with RV pacing under 50% had better event-free survival than those who were not (log rank p = 0.047). RV; right ventricle. [file Image_1.tif]

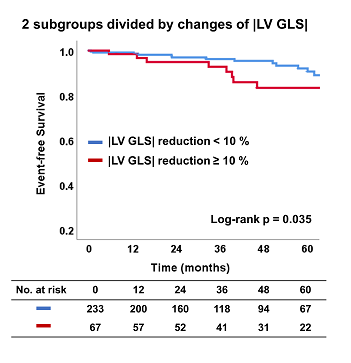

Supplement: Supplementary Figure 2 — Kaplan-Meier curve on 2 subgroups according to the degree of |LV-GLS| change between baseline and post-PM echocardiogram. The subgroup with the |LV-GLS| reduction under 10% had better event-free survival than those who were not (log rank p = 0.035). |LV-GLS|; absolute value of left ventricular global longitudinal strain. [file Image_2.tif]
